# Survey of women’s report for 33 maternal and newborn indicators: EN-BIRTH multi-country validation study

**DOI:** 10.1186/s12884-020-03425-6

**Published:** 2021-03-26

**Authors:** Shafiqul Ameen, Abu Bakkar Siddique, Kimberly Peven, Qazi Sadeq-ur Rahman, Louise T. Day, Josephine Shabani, Ashish KC, Dorothy Boggs, Donat Shamba, Tazeen Tahsina, Ahmed Ehsanur Rahman, Sojib Bin Zaman, Aniqa Tasnim Hossain, Anisuddin Ahmed, Omkar Basnet, Honey Malla, Harriet Ruysen, Hannah Blencowe, Fred Arnold, Jennifer Requejo, Shams El Arifeen, Joy E. Lawn, EN-BIRTH Study Group, Qazi Sadeq-ur Rahman, Ahmed Ehsanur Rahman, Tazeen Tahsina, Sojib Bin Zaman, Shafiqul Ameen, Tanvir Hossain, Abu Bakkar Siddique, Aniqa Tasnim Hossain, Tapas Mazumder, Jasmin Khan, Taqbir Us Samad Talha, Rajib Haider, Md. Hafizur Rahman, Anisuddin Ahmed, Shams El Arifeen, Omkar Basnet, Avinash K. Sunny, Nishant Thakur, Regina Gurung, Anjani Kumar Jha, Bijay Jha, Ram Chandra Bastola, Rajendra Paudel, Asmita Paudel, Ashish KC, Nahya Salim, Donat Shamba, Josephine Shabani, Kizito Shirima, Menna Narcis Tarimo, Godfrey Mbaruku, Honorati Masanja, Louise T. Day, Harriet Ruysen, Kimberly Peven, Vladimir Sergeevich Gordeev, Georgia R. Gore-Langton, Dorothy Boggs, Stefanie Kong, Angela Baschieri, Simon Cousens, Joy E. Lawn

**Affiliations:** 1 Maternal and Child Health Division, International Centre for Diarrhoeal Disease Research, Bangladesh (icddr,b), 68 Shahid Tajuddin Ahmed Sarani, Mohakhali, Dhaka, Bangladesh; 2 Centre for Maternal, Adolescent, Reproductive & Child Health (MARCH), London School of Hygiene & Tropical Medicine, London, UK; 3 Florence Nightingale Faculty of Nursing, Midwifery & Palliative Care, King’s College London, London, UK; 4 Department of Health Systems, Impact Evaluation and Policy, Ifakara Health Institute (IHI), Dar es Salaam, Tanzania; 5 International Maternal and Child Health, Department of Women’s and Children’s Health, Uppsala University, Uppsala, Sweden; 6 Research Division, Golden Community, Lalitpur, Nepal; 7 Demographic and Health Survey Program, ICF, Rockville, MD USA; 8 Division of Data, Analysis, Planning and Monitoring, United Nations Children’s Fund, Headquarters, New York, New York, USA

**Keywords:** Birth, Maternal, Newborn, Coverage, Validity, Survey, Indicators, Accuracy

## Abstract

**Background:**

Population-based household surveys, notably the Demographic and Health Surveys (DHS) and Multiple Indicator Cluster Surveys (MICS), remain the main source of maternal and newborn health data for many low- and middle-income countries. As part of the *Every Newborn* Birth Indicators Research Tracking in Hospitals (EN-BIRTH) study, this paper focuses on testing validity of measurement of maternal and newborn indicators around the time of birth (intrapartum and postnatal) in survey-report.

**Methods:**

EN-BIRTH was an observational study testing the validity of measurement for selected maternal and newborn indicators in five secondary/tertiary hospitals in Bangladesh, Nepal and Tanzania, conducted from July 2017 to July 2018. We compared women’s report at exit survey with the gold standard of direct observation or verification from clinical records for women with vaginal births. Population-level validity was assessed by validity ratios (survey-reported coverage: observer-assessed coverage). Individual-level accuracy was assessed by sensitivity, specificity and percent agreement. We tested indicators already in DHS/MICS as well as indicators with potential to be included in population-based surveys, notably the first validation for small and sick newborn care indicators.

**Results:**

33 maternal and newborn indicators were evaluated. Amongst nine indicators already present in DHS/MICS, validity ratios for baby dried or wiped, birthweight measured, low birthweight, and sex of baby (female) were between 0.90–1.10. Instrumental birth, skin-to-skin contact, and early initiation of breastfeeding were highly overestimated by survey-report (2.04–4.83) while umbilical cord care indicators were massively underestimated (0.14–0.22). Amongst 24 indicators not currently in DHS/MICS, two newborn contact indicators (kangaroo mother care 1.00, admission to neonatal unit 1.01) had high survey-reported coverage amongst admitted newborns and high sensitivity. The remaining indicators did not perform well and some had very high “don’t know” responses.

**Conclusions:**

Our study revealed low validity for collecting many maternal and newborn indicators through an exit survey instrument, even with short recall periods among women with vaginal births. Household surveys are already at risk of overload, and some specific clinical care indicators do not perform well and may be under-powered. Given that approximately 80% of births worldwide occur in facilities, routine registers should also be explored to track coverage of key maternal and newborn health interventions, particularly for clinical care.

## Key findings

**Table:** 

**What is known and what is new about this study?** • Population-based household surveys are the primary source of maternal and newborn health data for many low- and middle-income countries (LMICs). While surveys are important data sources, especially where coverage of robust routine health data systems remains low, they are also infrequent, expensive and have been shown to have limited validity for some aspects of perinatal care. • EN-BIRTH is the largest validation study to date of maternal and newborn health indicators, across five hospitals in three countries, including > 14,000 exit surveys. This dataset enabled validity analyses to be made for 33 maternal and newborn indicators comparing gold-standard observation or case notes verification to exit survey-reported coverage and outcomes. • This is the first validity testing for hospital-based clinical care of small and sick newborns (e.g. resuscitation, kangaroo mother care, and neonatal infection management). **What does this say about nine indicators already in MICS and/or DHS core or additional modules?** • 4 out of 9 indicators were accurately estimated by survey report at the pooled population level: baby dried or wiped immediately after birth (observed coverage: 90.5%, survey: 96.8%), birthweight measured (observed: 98.6%, survey: 93.8%), sex (observed female: 48.8%, survey-reported female: 49.1%) and low birthweight (based on observed weight: 15.2%, based on survey-reported weight: 14.1%). • Early initiation of breastfeeding (observed: 14.4%, survey: 69.5%), and skin-to-skin contact (observed: 41.2%, survey: 84.2%) were highly overestimated in the exit survey. • Application of a substance to the umbilical cord was massively (> 76%) underestimated in survey-report compared with observed coverage for anything applied to the cord or chlorhexidine applied to the cord, largely driven by “don’t know” responses (24.1–75.2%). • Besides application of a substance/chlorhexidine to the umbilical cord, “don’t know” responses were < 10% for other indicators already in DHS/MICS. **Which questions are not appropriate for surveys?** • Validity of indicators not already in DHS/MICS was affected by high “don’t know” responses (> 20%), varying widely by hospital e.g. birth attendant listened to fetal heart sounds during labour, oxytocin given, antenatal corticosteroids given before birth, baby received injectable antibiotics, any diagnostic/blood test done. • Clinical care indicators had low validity: any infection (percent agreement 47.1%) or sepsis (percent agreement 26.6%). Newborn resuscitation had high percent agreement and high specificity but low sensitivity. Moreover, indicators for the small and sick newborn target group may be underpowered even in large national household surveys. **What next and research gaps?** • Consistent with other studies, we found lower validity for clinical interventions and time-bound questions. Further research is needed on time-bound indicators to explore how the accuracy of crucial indicators such as early initiation of breastfeeding can be improved, e.g. if the time component were to be dropped. • Women whose newborns were admitted to a neonatal or KMC ward reported this accurately; however, to be useful in population-based surveys, we would need to know how people not admitted would respond. • Improved, respectful communication with families regarding clinical interventions for small and sick newborns is needed for both quality care and accuracy of survey-reported coverage. Families cannot report on clinical care if they were never informed.	

## Background

Globally each year, 2.4 million newborns die in the first month of life, more than 2 million babies are stillborn and around 295,000 women die of maternal causes, the vast majority in low- and middle-income countries (LMICs) [1–4]. Most of these deaths can be prevented by high coverage and quality care during pregnancy and childbirth, and for small and sick newborns [5]. The Sustainable Development Goals include a target to reduce the national neonatal mortality rate to fewer than 12 per 1000 live births, and the global average maternal mortality ratio to fewer than 70 per 100,000 live births by 2030 [6]. To track the progress, and the linked stillbirth target of fewer than 12 stillbirths per 1000 total births, the *Every Newborn* Action Plan (ENAP) was launched in 2014. In close alignment with the World Health Organization Strategy for Ending Preventable Maternal Mortality, some indicators were prioritised for maternal and newborn care [7, 8]. Unfortunately, in the countries where most of the maternal and newborn deaths occur, data gaps for coverage and quality of care impede health systems improvement needed to drive progress towards universal health coverage [9].

Currently, most LMICs are reliant on retrospective data based on women’s self-report collected through household surveys, such as the Demographic and Health Surveys (DHS) and Multiple Indicator Cluster Surveys (MICS) [10, 11]. However, these population-level surveys track a limited number of indicators that measure maternal and newborn care and have focused especially on “contact points” such as antenatal care, skilled birth attendance, facility birth, and postnatal care. The DHS core women’s questionnaire has over 400 questions and takes 30–60 min to complete for most women; there is understandable reluctance to add more questions on maternal and newborn health.

Given the shift to evidence-based measurement, there is more demand for validation studies on indicators already in surveys, and to inform selection of new questions. It is recommended to test the validity by comparing survey measures of an indicator to a gold standard data source [12]. Several studies have assessed validity of women’s self-report in high income or upper middle-income countries using clinical records as the gold standard [13–26]. Other studies have sought to validate women’s reports of events related to care around the time of birth in LMICs using direct observation as the gold standard; however, these studies often had a small sample size and/or were conducted in one or two facilities [27–30]. These studies have found variable validity for some indicators including uterotonic administration, early initiation of breastfeeding and skin-to-skin contact, indicating need for further research in additional contexts. Previous validity studies have not been conducted in Bangladesh or Tanzania and those in Nepal have been limited to birthweight and gestational age [31]. Furthermore, no published studies have reported on validity of women’s reports for new indicators related to the care of small and sick newborns such as kangaroo mother care (KMC) for low birthweight babies or injectable antibiotics for newborn sepsis.

The *Every Newborn* Action Plan, agreed by all United Nations member states and > 80 development partners, includes an ambitious measurement improvement roadmap to validate measurement of indicators for care and outcomes around the time of birth [7, 32]. As part of this roadmap, the *Every Newborn* – Birth Indicators Research Tracking in Hospitals (EN-BIRTH) study was an observational study of > 23,000 women to test the validity of measurement for selected indicators [33].

## Objectives

This paper is part of a supplement based on the EN-BIRTH multi-country validation study, *‘Informing measurement of coverage and quality of maternal and newborn care’* and focuses on women’s report surveys, with the following objectives:

1. **Assess the VALIDITY of measurement for nine current DHS/MICS indicators** that measure care during the intrapartum and immediate postpartum period for women with vaginal births.
2. **Explore 24 POTENTIAL MATERNAL AND NEWBORN INDICATORS,** including indicators for care of small and sick newborns that could be included in population surveys (e.g. DHS/MICS) and assess their validity and measurement quality.

## Methods

### Study settings and design

**Fig. 1:**
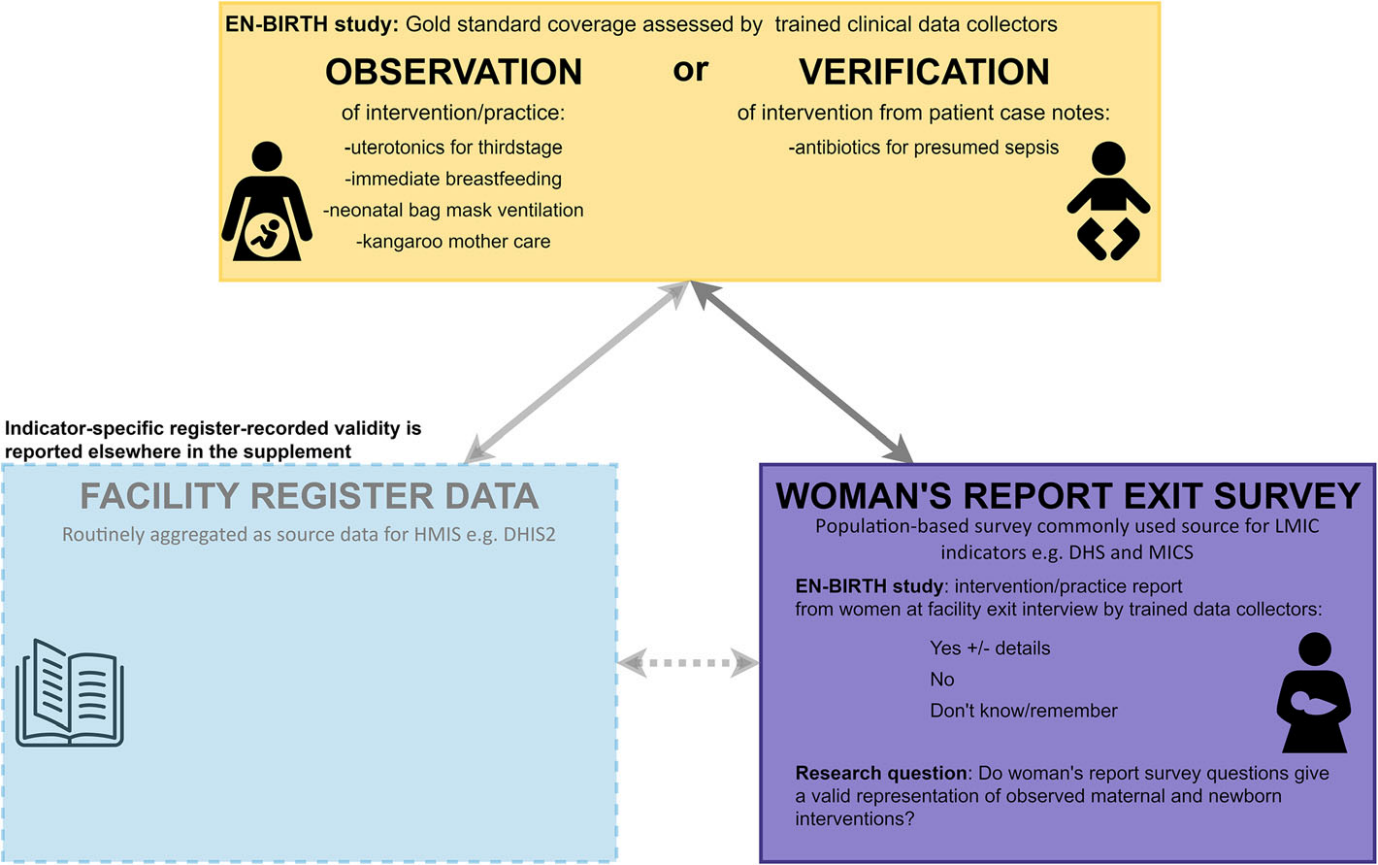
Survey validation design, EN-BIRTH study. Exact wording for survey questions detailed in Additional file 1

EN-BIRTH was a mixed-methods, observational study comparing observer-assessed coverage (considered the gold standard) of selected maternal and newborn interventions to coverage measured by women’s report at exit survey and routine register records; this paper focuses on survey-report (Fig. 1). Data were collected from July 2017 to July 2018 in five public secondary/tertiary comprehensive emergency obstetric and newborn care (CEmONC) hospitals in three high burden countries: Maternal and Child Health Training Institute, Azimpur and Kushtia General Hospital in Bangladesh (BD); Pokhara Academy of Sciences in Nepal (NP); Temeke District Hospital and Muhimbili National Referral Hospital in Tanzania (TZ). Detailed information regarding the research protocol [33] and overall validity results, including for routine register data, has been published separately [34].

Participants were pregnant women being admitted to labour/delivery wards (exclusion criteria at admission were imminent birth and no fetal heart beat heard, participants were not automatically excluded based on age), mother-baby pairs admitted in KMC corners/wards (all admissions were eligible) and newborns admitted to inpatient wards for treatment of presumed severe neonatal infection (neonates with clinically defined infection—sepsis, pneumonia, meningitis—were eligible). Trained clinical researchers observed participants 24 h per day and recorded data on care and outcomes in three clinical settings: labour and delivery ward, operating theatre, and KMC corners/wards. Verification of inpatient records were used as the gold standard for newborns who received antibiotics for presumed severe infection, and for women who received antenatal corticosteroids (ACS) for risk of preterm birth. Women were surveyed at the time of discharge before leaving the facility by a separate cadre of data collectors. Training for survey data collectors was based on DHS training materials. In the case of multiple births, women were asked only about the first birth. All data were collected using a custom-built android tablet-based application. All data collectors and study staff received standardised training on the study procedures and data collections tools.

We compared observer-assessed coverage of care and outcomes for women with vaginal births to women’s reports at exit survey. Women who give birth vaginally on labour wards have a very different experience than those giving birth by caesarean in operating theatre; thus we have analysed these separately. Differences between vaginal birth and caesarean birth for indicator accuracy are reported elsewhere for five indicators [34] and for specific indicators throughout this supplement series [35–38]. Further work is ongoing to examine quality of care and measurement accuracy for women with caesarean sections.

### Indicator selection

**Fig. 2:**
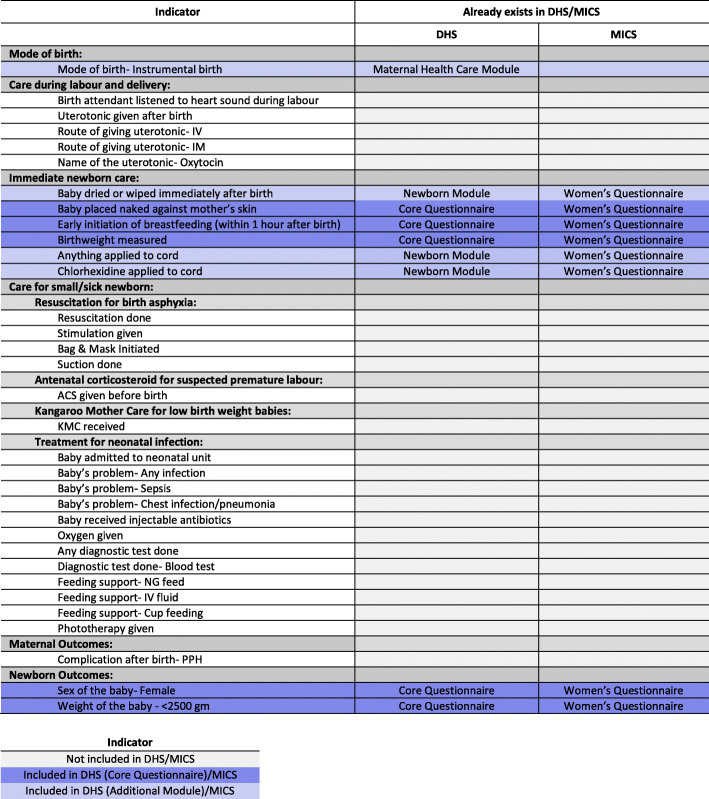
List of indicators tested for validity, EN-BIRTH study

At the study design phase, we conducted a mapping review of the MICS women’s questionnaire as well as DHS-7 core women’s questionnaire and newborn care additional module to identify maternal and newborn indicators from the intrapartum and immediate postnatal period for which we could test validity in a hospital setting. To identify maternal and newborn indicators that have the potential to be included in population-based surveys, we referred to the Ending Preventable Maternal Mortality, *Every Newborn* Action Plan strategy documents and earlier studies testing validity of measurement for maternal and newborn indicators [7, 8]. As a result of updates to DHS questionnaires (DHS-8) and the new supplemental module on maternal health care, we conducted an additional mapping review prior to data analysis. We selected 33 maternal and newborn indicators for analysis (nine indicators already existing in DHS/MICS and 24 indicators having the potential to be included in population-based surveys). The indicator list is shown in Fig. 2 and the exact wording for questions in EN-BIRTH, DHS and MICS is shown in Additional file 1.

### Analysis

To calculate observer-assessed coverage and survey-reported coverage, we used a relevant denominator from the EN-BIRTH dataset (total deliveries/ total births/ livebirths/ admitted to KMC ward/ admitted to inpatient for suspected neonatal infection, etc.) (Additional file 2) and expressed results as a percentage. “Don’t know” survey responses were also reported separately as a percentage. We calculated validity ratios, similar to verification ratios in data quality review (DQR) methods, calculating survey-coverage divided by observed coverage where “don’t know” responses were treated as “no” (Additional file 3). A ratio > 1 shows overestimation of survey-reported coverage compared to observed, while a ratio < 1 shows an underestimate. We used standard DQR cut-offs (over/underestimate by 0–5% = Excellent, by 6–10% = Very good, by 11–15% = Good by, 16–20% = Moderate and by  > 20% = Poor) for heat maps [39].

For individual-level validity reporting, we constructed two-way tables comparing observer-assessed coverage to survey-reported coverage. In line with DHS and common survey reporting, we combined survey “don’t know” responses with “no”, except for the low birthweight indicator where “don’t know” was excluded from the numerator and denominator [40]. Additional analysis for selected indicators is presented in Additional file 4 with “don’t know” excluded from the analysis (numerator and denominator).

As interventions/conditions with very high or very low coverage/prevalence may result in a small sample size for individual-level validity “diagnostic test” methods (low cell counts in two way tables), we report percent agreement for all indicators. Where column totals are ≥10 in the two way tables and “don’t know” responses were < 20%, we calculated sensitivity (true positive rate) and specificity (true negative rate) of survey-reported coverage to measure observed coverage (gold standard). Positive predictive value (PPV), negative predictive value (NPV), area under the curve (AUC), and inflation factor (IF) were also calculated. Percentage observed to have an intervention or outcome among women replying “don’t know” for indicators included in DHS/MICS were calculated. 95% confidence intervals were calculated assuming a binominal distribution. Validity analysis pooled results were calculated using random effects meta-analysis, presented with i^2^, τ^2^, and heterogeneity statistic (Q). Missing values from the observation dataset were excluded from the relevant analysis [12].

To determine reliability of the observational data (gold standard), study supervisors simultaneously observed births with data collectors for a 5% subset of cases. We calculated percent agreement and Cohen’s kappa coefficients of agreement for core indicators. Percent agreement between the two observers ranged between 85.0–100% by indicator and site. Kappa scores had a wider range (0–1), however, some low kappa scores were affected by prevalence and an imbalance in marginal totals. We included all indicators in the analysis and discussion on low kappa coefficients has been done elsewhere [34].

All statistical analyses were conducted using Stata (version 16) [41] and results are reported in accordance with STROBE statements checklists for cross-sectional studies (Additional file 5) [42].

## Results

### Study participants

**Fig. 3:**
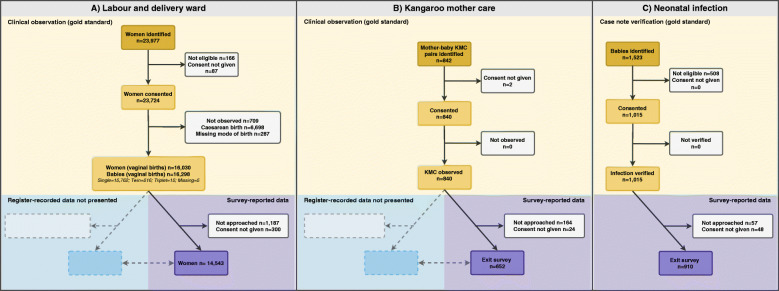
Flow Diagram: **a** Labour and Delivery **b** Kangaroo Mother Care **c** Neonatal Infection, EN-BIRTH study

Three types of participants were involved in this study (Fig. 3). Amongst 23,015 women observed in labour and delivery wards, 16,030 had vaginal births resulting in 16,298 newborns (including twins and stillbirths). Exit surveys were conducted with 14,543 of these women (90.7%). Out of 842 mother-baby pairs admitted in KMC wards/corners, 840 pairs were observed (99.8%). Exit surveys were conducted with 652 women (77.6%). A total of 1523 babies were identified in the inpatient wards, 1015 met eligibility criteria for presumed severe infection (diagnosis of sepsis, pneumonia, or meningitis), consent was obtained for 100% and exit surveys were conducted with 910 women (89.7%). Reasons for non-participation in the exit survey included refusal and discharge prior to being approached for the survey.

The background characteristics of the participants are presented in Table 1, with details by site in Additional files 6, 7 and 8. One-third (37.3%) were age 20–24, more than 40% completed secondary education and half (51.1%) were pregnant for the first time. Among the babies who were observed in the KMC ward, 97.0% were less than or equal to 2000 g. Among the babies who were admitted in the inpatient ward for presumed severe infection and met the eligibility criteria, two-thirds were less than 7 days old at the time of admission.

**Table 1:** Characteristics of women and babies, EN-BIRTH study

Characteristics	Labour & Delivery (All sites)	Kangaroo Mother Care (All sites)	Neonatal Infection (All sites)
**Total women**	**16,030**	**840**	**1015**
**Woman’s age**			
< 18 years	318 (2.0)	30 (3.6)	26 (2.6)
18–19 years	1803 (11.2)	103 (12.3)	128 (12.6)
20–24 years	5978 (37.3)	292 (34.8)	352 (34.7)
25–29 years	4371 (27.3)	211 (25.1)	285 (28.1)
30–34 years	2338 (14.6)	132 (15.7)	151 (14.9)
35+ years	1222 (7.6)	72 (8.6)	73 (7.2)
**Woman’s education**			
No education	503 (3.1)	34 (4)	34 (3.3)
Primary incomplete	414 (2.6)	16 (1.9)	36 (3.5)
Primary complete	584 (3.6)	38 (4.5)	79 (7.8)
Secondary incomplete	6437 (40.2)	399 (47.5)	400 (39.4)
Secondary complete or higher	7644 (47.7)	340 (40.5)	436 (43.0)
Don’t know	448 (2.8)	13 (1.5)	30 (3.0)
**Birth order**			
Nullipara	8199 (51.1)	0 (0.0)	0 (0.0)
Primipara	4606 (28.7)	421 (50.1)	627 (61.8)
Multipara (2–4)	3062 (19.1)	341 (40.6)	344 (33.9)
Grand Multipara (5+)	126 (0.8)	77 (9.2)	38 (3.7)
Don’t know	34 (0.2)	1 (0.1)	6 (0.6)
Missing	3 (0.0)	0 (0.0)	0 (0.0)
**Mode of birth**			
Normal vertex birth	15,661 (97.7)	–	–
Vaginal breech	93 (0.6)	–	–
Vacuum/Forceps	276 (1.7)	–	–
**Baby Total**	**16,298**	**840**	**1015**
**Sex**			
Male/Boy	11,902 (50.7)	379 (45.1)	624 (61.5)
Female/Girl	11,042 (47)	458 (54.5)	391 (38.5)
Ambiguous	31 (0.1)	1 (0.1)	0 (0.0)
Missing	496 (2.1)	2 (0.2)	0 (0.0)

All sites, unweighted

### Indicators already captured in MICS/DHS

**Fig. 4:**
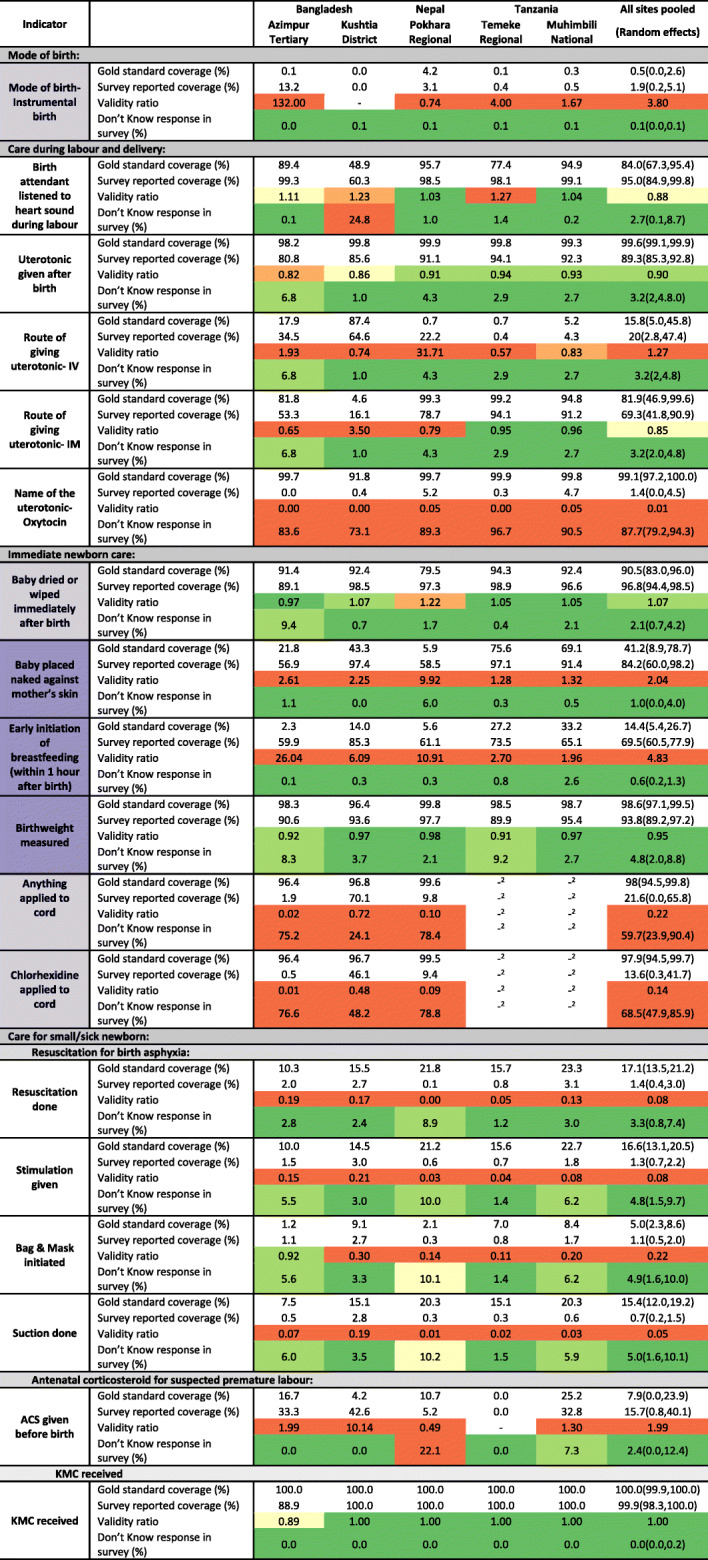

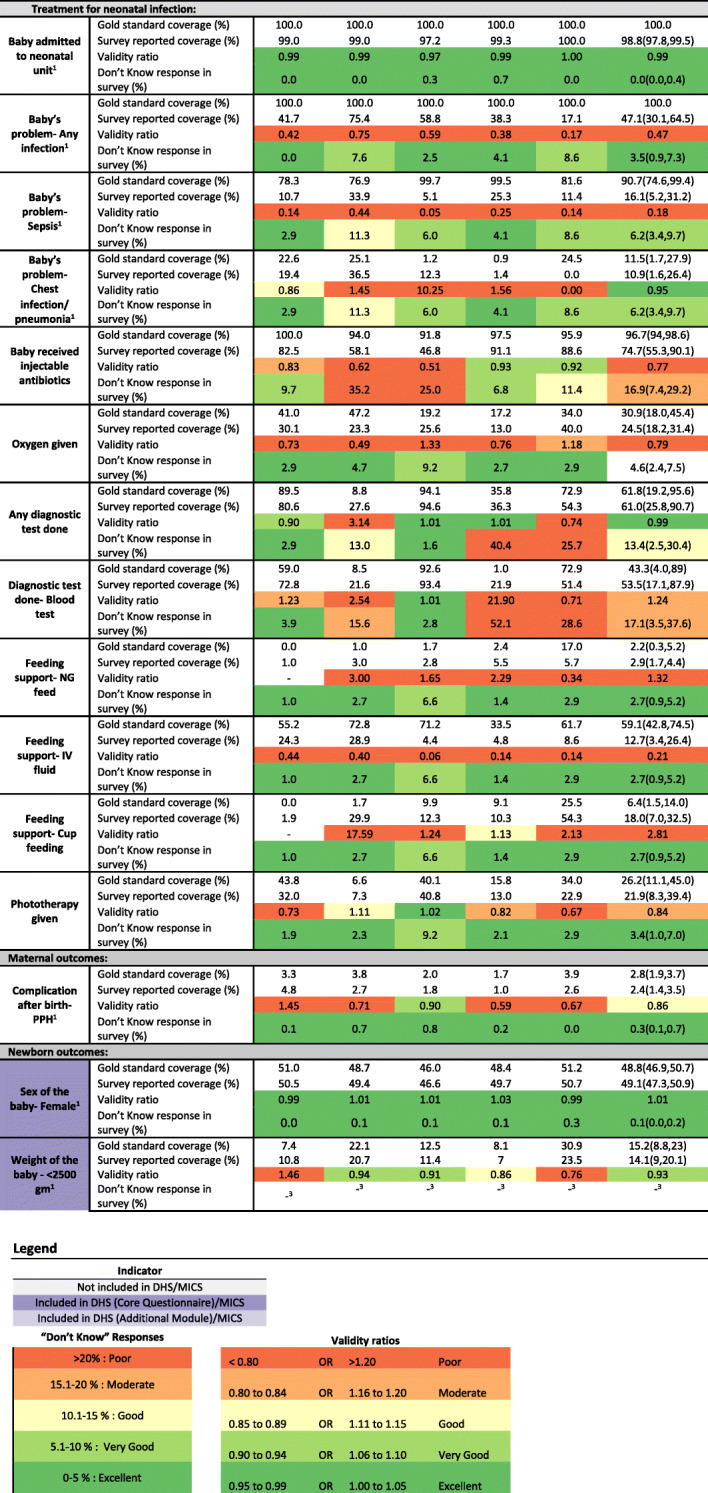
Coverage for selected indicators, EN-BIRTH study. ^1^These indicators are not interventions and prevalence is reported for these indicators. ^2^Not asked in Tanzania. ^3^"Don’t Know" is excluded from numerator and denominator. Validity ratio calculated as survey-coverage/observed coverage. Observed data: labour and delivery ward *n* = 16,030 women, 16,298 newborns; kangaroo mother care *n* = 840; neonatal infection *n* = 1015. Survey data: labour and delivery ward *n* = 14,543 women; kangaroo mother care *n* = 652; neonatal infection *n* = 910

**Fig. 5:**
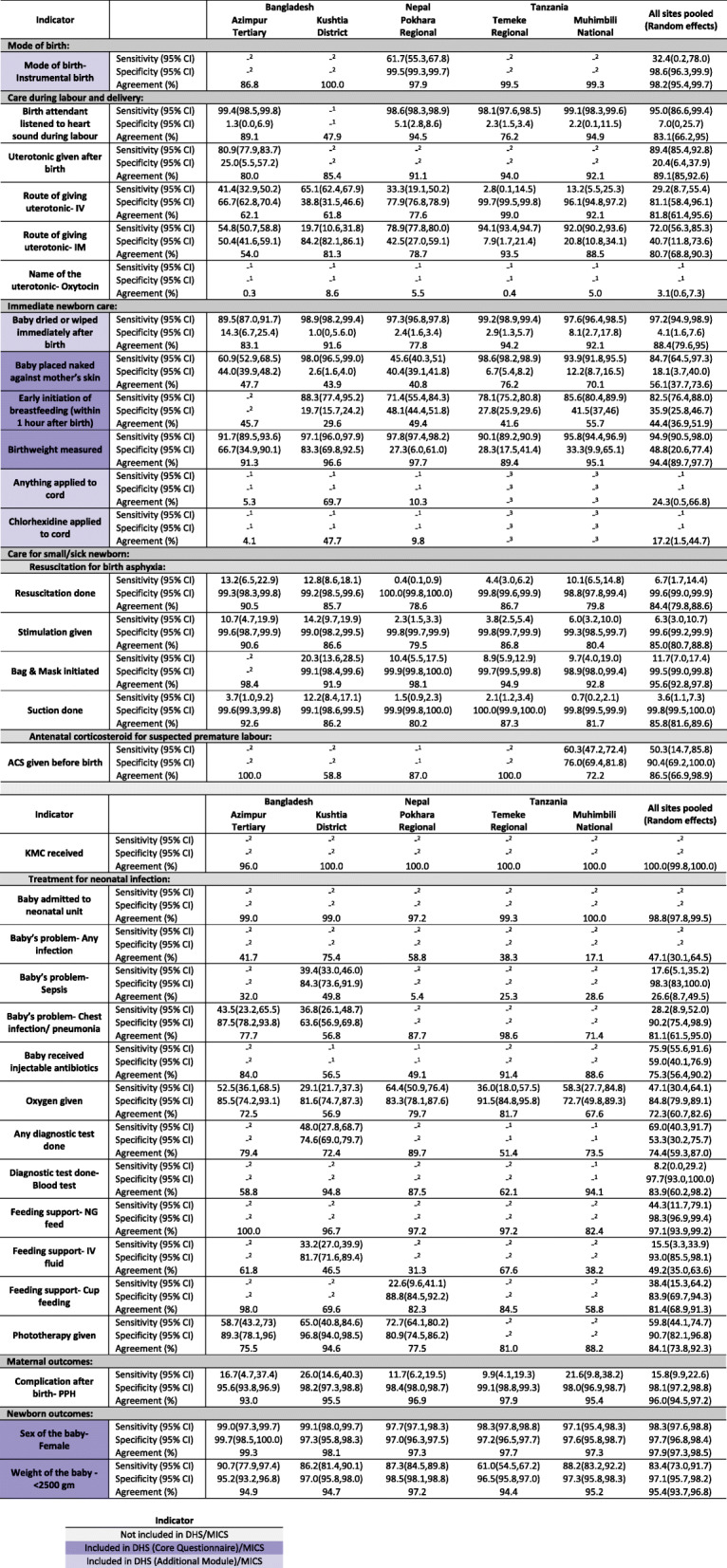
Individual-level validation in exit survey for selected indicators, EN-BIRTH study. ^1^Validation not done because “Don’t Know” response > 20%. ^2^Validation not done because ten or fewer observations per column of the two-way table. ^3^Not asked in Tanzania. Observed data: labour and delivery ward *n* = 16,030 women, 16,298 newborns; kangaroo mother care *n* = 840; neonatal infection *n* = 1015. Survey data: labour and delivery ward *n* = 14,543 women; kangaroo mother care *n* = 652; neonatal infection *n* = 910

Four out of nine indicators were accurately estimated by survey report at the pooled population level. Indicators with a time component (early breastfeeding and skin-to-skin contact) were over-reported while umbilical cord care indicators were under-reported. Instrumental birth had very low coverage and was overreported in survey. Figure 4 and Additional file 9 present the observer-assessed coverage and survey-reported coverage along with the percentage of “don’t know” responses. Sensitivity, specificity, and percent agreement are presented in Fig. 5 and Additional files 10 and 11.

Mode of birth (instrumental birth) had very low “don’t know” responses across all hospitals (< 1%, Fig. 4, Additional file 9). Observed coverage of instrumental birth was low (0.5%) while survey reported coverage was 1.9%. While percent agreement was 98.2% the validity ratio was “poor” (3.80) (Fig. 5). Individual level validity statistics could only be calculated for Pokhara NP due to low cell counts in two-way tables and showed low sensitivity (61.7%) and high specificity (99.5%, Additional file 10).

Immediate newborn care indicators ranged in coverage and performance. Immediate drying and birthweight measured had high observed coverage (> 90%) and low levels of “don’t know” responses (< 5%). While sensitivity was high (> 94%), specificity was low (4.1% for immediate drying, 48.8% for birthweight measured) and validity ratios were classified as “very good” (immediate drying: 1.07) and “excellent” (birthweight measured: 0.95). Early initiation of breastfeeding and skin-to-skin contact were both largely overestimated by the survey despite low levels of “don’t know” responses (< 1%). Observed coverage of early initiation of breastfeeding was very low (< 14.4%), while survey-reported coverage was 69.5%. While sensitivity was high (82.5%), specificity was low (35.9%) and validity ratio was “poor” (4.83). Observed coverage of skin-to-skin contact was 41.2%, while survey-reported coverage was 84.2%. Sensitivity was high (84.7%) while specificity was low (18.1%) and validity ratio was “poor” (2.04). Cord cleansing with chlorhexidine was nearly universal (97.9%) in the three hospitals with a chlorhexidine policy (Bangladesh and Nepal). Survey-reported coverage, however, had a large range. While survey-reported coverage of any cord cleansing and chlorhexidine application was very low in Azimpur BD (1.9% and 0.5%, respectively) survey-reported coverage for these interventions was higher in Kushtia BD (70.1% and 46.1%, respectively). Overall, sensitivity was low (21.7% for anything applied to the cord; 13.9% chlorhexidine) and specificity was higher (79.1% anything applied; 92.6% chlorhexidine). Validity ratios were “poor” (0.22 and 0.14).

Newborn outcomes such as sex of the baby and low birthweight were well estimated by the survey. Sex of the baby had very low “don’t know” responses (< 1%) with high sensitivity, high specificity (> 97%) and “excellent” validity ratio (1.01). Women were asked the birthweight of the baby, which was then categorized into low (< 2500 g) or normal birthweight (≥2500 g). “Don’t know” responses to birthweight were moderate (4.8%). For low birthweight classification, sensitivity was 83.4%, specificity was 97.1% and validity ratio was “very good” (0.93).

### “Don’t know” responses analysis considerations

Among women who replied “don’t know” in the survey, the proportion observed to have the intervention or outcome is presented in Table 2. Of those who didn’t know if their baby was dried or wiped immediately after birth, most (79.2%) were observed to be dried/wiped. Similarly, for women who didn’t know about birthweight measurement or cord care practices, most were observed as completed (birthweight measured: 91%, anything applied to cord: 97.4%, Chlorhexidine applied to cord: 97.2%). However, for interventions involving women themselves, such as placing the newborn skin-to-skin or initiation of breastfeeding, observed coverage among women responding “don’t know” in survey was low.

**Table 2:** Percentage observed to have intervention/outcome despite reporting “don’t know” for indicators included in DHS/MICS, EN-BIRTH study

Indicator		Bangladesh		Nepal	Tanzania		All sites pooled and 95%CI (Random effects)
		Azimpur Tertiary	Kushtia District	Pokhara Regional	Temeke Regional	Muhimbili National	
**Mode of birth:**							
**Mode of birth- Instrumental birth**	% Observed as instrumental birth	–	0.0	33.3	0.0	0.0	2.8 (0.0,34.3)
	Total “Don’t Know” Responses in Survey	0	2	3	4	1	10
**Immediate newborn care:**							
**Baby dried or wiped immediately after birth**	% Observed dried/wiped	87.1	88.9	73.5	76.2	68.2	79.2 (70.5,86.7)
	Total “Don’t Know” Responses in Survey	70	9	98	21	22	220
**Baby placed naked against mother’s skin**	% Observed skin-to-skin	25.0	–	17.1	37.5	100.0	41.4 (11.3,75.0)
	Total “Don’t Know” Responses in Survey	8	0	346	16	5	375
**Early initiation of breastfeeding (within 1 h after birth)**	% Observed breastfeeding within 1 h	0.0	25.0	0.0	11.6	11.1	3.0 (0.0,10.5)
	Total “Don’t Know” Responses in Survey	1	4	15	43	27	90
**Birthweight measured**	% Observed weighed	91.9	64.0	95.9	97.3	93.3	91.0 (80.5,97.9)
	Total “Don’t Know” Responses in Survey	62	50	123	490	30	755
**Anything applied to cord**	% Observed anything applied to cord	96.2	95.6	99.3	−^1^	−^1^	97.4 (93.6,99.6)
	Total “Don’t Know” Responses in Survey	555	315	4485	−^1^	−^1^	5353
**Chlorhexidine applied to cord**	% Observed chlorhexidine applied to cord	95.9	95.5	99.1	−^1^	−^1^	97.2 (93.5,99.4)
	Total “Don’t Know” Responses in Survey	565	629	4508	−^1^	−^1^	5700
**Newborn outcomes:**							
**Sex of the baby- Female**	% Observed female	–	0.0	71.4	40.0	66.7	56.4 (25.9,85.2)
	Total “Don’t Know” Responses in Survey	0	1	7	5	3	16
**Weight of the baby - < 2500 g**	% classified low birthweight (< 2500 g)	6.4	8.3	22.1	8.0	46.1	16.1 (6.6,28.8)
	Total “Don’t Know” Responses in Survey	110	96	308	1097	102	1713

1 = Not asked in Tanzania

For indicators included in DHS/MICS

**Table 3:** Estimated sample size required to measure coverage of kangaroo mother care in a national household survey

Households needed	Bangladesh	Nepal	Tanzania
**5% Coverage**	356,197	220,963	268,266
**10% Coverage**	168,725	104,667	127,074
**25% Coverage**	56,242	34,889	42,358
**50% Coverage**	18,747	11,630	14,119
**75% Coverage**	6249	3877	4706
**Last DHS survey**	19,457	11,040	12,563

$$ \boldsymbol{n}=\frac{\mathbf{4}\left(\boldsymbol{r}\right)\left(\mathbf{1}-\boldsymbol{r}\right)\left(\boldsymbol{f}\right)\left(\mathbf{1}.\mathbf{1}\right)}{\left(\mathbf{0.12}{\boldsymbol{r}}^{\mathbf{2}}\right)\left(\boldsymbol{p}\right)\left({\boldsymbol{n}}_{\boldsymbol{h}}\right)} $$

where:***r****=* predicted coverage

***f*** *=* design effect = 1.5

***p***= population proportion of target group (under 5 years and under 2000 g)

***n***_***h***_= household size

Validity results with “don’t know” responses excluded are shown in Additional file 4. When “don’t know” responses were excluded for anything applied to the cord and chlorhexidine applied to the cord, individual-level validity improved. Other indicators had low “don’t know” responses and little change to validity when these were excluded.

### Indicators not currently in DHS/MICS

#### Contact point coverage indicators with potential for surveys

Survey measurement was tested for two contact indicators for small and sick newborns, validity ratios were “excellent” among admitted newborns. Among women whose newborns were admitted to a neonatal unit for treatment of infection, 98.8% reported their newborns were admitted to a neonatal unit, and “don’t know” responses were < 1%. Women whose newborns were not admitted and did not have an infection diagnosis were not asked about admission to a neonatal unit. Similarly, both observed and survey reported coverage of KMC among women whose newborns were admitted to KMC corners/wards was universal. Percent agreement was 100%, however this must be interpreted with caution due to 100% coverage.

#### Content indicators with limited potential for surveys

Of the remaining 22 content indicators, seven had validity ratios of “good” or better when pooled across sites, however none were consistently good across all sites. Questions related to clinical interventions during labour and childbirth, such as listening to fetal heart sounds or administration of uterotonics, did not perform consistently well in the survey. While listening to fetal heart sounds had high sensitivity (> 98%), specificity was very low, < 6%. Questions around uterotonics had variable percent agreement, sensitivity and specificity.

Observed coverage of any resuscitation (stimulation, suction, bag-mask-ventilation (BMV)) was 17.1% where coverage was highest for stimulation (16.6%) and BMV coverage was 5%. Survey-reported stimulation was 1.3%, underestimating observed stimulation by 15.3 percentage points (validity ratio: 0.08). While specificity was high (> 99%) sensitivity was less than 7%. Similarly, suction and BMV were overestimated by surveys and had high specificity (> 99%), low sensitivity (< 12%), and “poor” validity ratios (< 0.22).

Among women whose newborns were admitted to a neonatal unit for infection, very few were able to report that their baby had an infection, and survey-reported prevalence underestimated verified prevalence by 24.6–82.9 percentage points with a “poor” validity ratio (0.47). Survey-reported receipt of injectable antibiotics was under-estimated in surveys by 6.4–45.0 percentage points, and “don’t know” responses ranged from 9.7–35.2% (validity ratio: 0.77). Patient notes verified oxygen administration ranged from 17.2–47.2% and survey-report ranged from underestimating oxygen administration by 6.4 percentage points to overestimating it by 23.9 percentage points (validity ratio: 0.79). “Don’t know” responses for diagnostic testing ranged from 1.6–40.4%. While in Pokhara NP there were few “don’t know” responses (1.6%) and survey-reports were very close to notes of verified coverage (within 1%), in Muhimbili TZ “don’t know” responses were 25.6% and verified coverage was underestimated by 18.6 percentage points.

Amongst women whose newborn was admitted to KMC corners/wards, while nasogastric feeding was low (0–17.0%) with a “poor” validity ratio (1.32), intravenous feeding support ranged from 55.2–72.8% and was underestimated by survey-report by 28.7–66.8 percentage points. Coverage of phototherapy ranged from 6.6–43.8% and was close to survey-reported coverage (validity ratio: 0.84).

Prevalence of postpartum haemorrhage ranged from 1.7–3.9% and had high specificity (> 95%) but low sensitivity (< 27%).

## Discussion

Currently, population-based surveys capture limited data on maternal and newborn care and few validity studies have evaluated available or potential indicators. EN-BIRTH study across five hospitals in three countries included > 14,000 women with vaginal births observed and with exit surveys, seven times more births than any previous maternal and newborn indicator validation study. Our dataset enabled validity analyses for measurement of 33 maternal and newborn indicators comparing time-stamped gold-standard observation to exit survey-reported indicators of coverage and outcomes, with nine indicators currently included in DHS/MICS and new indicators with potential for inclusion.

Overall, we found 4 of 9 indicators already in DHS/MICS performed well in surveys. Of indicators not already in DHS/MICS, “contact” indicators for small and sick newborns (admission to a neonatal unit or KMC ward) may be useful in population-based surveys while indicators on content of clinical care had high levels of “don’t know” responses and limited validity. Where previous validation research has shown mixed results, for example uterotonics for prevention of postpartum haemorrhage [26–30], we found survey report under-estimated true coverage by 10% whereas survey report overestimated early initiation of breastfeeding by nearly 5 times.

This is the first validity testing for hospital-based clinical care of small and sick newborns (e.g. resuscitation, KMC, and neonatal infection management). EN-BIRTH study allowed us to assess validity for these smaller number of vulnerable newborns who needed special care such as: neonatal resuscitation (5–10%) [43, 44], KMC for newborns weighing ≤2000 g (10–20%) [45, 46] and treatment of newborn presumed severe infection (7%) [47], which have not been validated before, partly because of sample size challenges, but also because policy attention is more recent [48]. Coverage of KMC was accurate by survey-report in our study although exit survey questions on KMC were asked only for those women whose newborns were admitted to a KMC ward. Further research is required to validate this indicator for all women, including those not admitted to a KMC ward. Population-based surveys, however, even when conducted with large sample sizes, may be under-powered to measure KMC targeted to stable newborns ≤2000 g. Sample size calculations suggest that for current levels of coverage of KMC for neonates ≤2000 g (believed to be under 10%), a national household survey in Nepal would need to have a 10-fold higher sample size than the most recent DHS survey (Table 3). Usefulness of surveys for interventions in subset target groups is a function of the prevalence of the clinical need for the intervention (i.e. denominator) and coverage, thus once KMC coverage reaches over 50%, then currently used national DHS sample size may suffice.

Indicators related to treatment for presumed severe neonatal infection, particularly those related to antibiotic treatment, may be difficult to capture through surveys. Among newborns admitted for treatment of presumed severe infection, we found poor validity in questions about the baby’s diagnosis and treatment, even with short recall periods. Previous studies of survey-reported antibiotic use for childhood illness have shown that these questions perform poorly and were even worse with longer recall periods [49]. These studies also found that maternal reports of symptoms of acute respiratory infection do not provide a correct denominator for monitoring antibiotic treatment rates [50].

Admission to a neonatal unit for infection may be a useful contact point indicator as women were able to report this with high sensitivity. However, similar to KMC, this exit survey question was only asked to women with admitted newborns and further research is required to validate this indicator in a wider population. Additionally, neonatal infection questions will be subject to sample size issues similar to KMC as incidence risk of possible severe bacterial infection is estimated at 7.6% [47]. Hospital registers and records may be a better alternative for reporting coverage of interventions for small target groups such as small and sick newborns. Specific registers can be designed for documentation of treatment of infection in neonatal inpatient wards rather than only maintaining individual case record forms [51].

For indicators already present in DHS/MICS, we found sex of the baby and low birthweight were reported accurately, although birthweight is known to have issues with heaping (preferential reporting of weight with numbers ending in 00) [35, 46]. Immediate drying had very high sensitivity but very low specificity, possibly relating to the timing element. Drying was counted as “immediate” when it was observed as done within 5 min of birth while women were asked, “Was your baby dried or wiped immediately after birth (within a few minutes)?”. In qualitative interviews with women about their understanding of the word "immediate" in questions relating to immediate newborn care, McCarthy et al. found a wide range of responses including 1 or 2 min, up to 7 min, and less than 20 min [30]. Other studies have also shown immediate drying to have high sensitivity, and low or moderate specificity alone or as a composite indicator with other immediate newborn care [27, 28, 30]. Similar to other validation studies, we found early initiation of breastfeeding was largely over-estimated by survey-reported coverage. This over-estimate may be due to poor recall of the timing component if breastfeeding was initiated but not within 1 h [26, 28, 29]. Furthermore, definitions of breastfeeding may differ between clinical observers and breastfeeding women. A woman may have put her baby to the breast and considered this initiation of breastfeeding, but an observer may not have recorded breastfeeding initiation if they did not observe attachment and suckling, as breastfeeding is a complex and dynamic process [34, 37]. Survey questions on breastfeeding may be more accurate if the focus on timing is removed or shifted to something easier to recall such as place.

While interventions involving women themselves, (e.g. skin-to-skin contact or initiation of breastfeeding) had low “don’t know” responses, questions regarding clinical interventions had high levels of “don’t know” responses. These indicators had lower accuracy in survey-reports, even when the recall period was very short (exit survey) compared with 2 to 5-year recall periods expected in population-based surveys. Low accuracy may relate to not seeing an intervention happening if newborns are separated from their mothers or may relate to poor communication about care from health care workers. While a study conducted in primary health care facilities in northern Nigeria found high validity for measurement of Chlorhexidine application to newborn’s cord [27], our study showed low validity in these facilities, possibly due to not applying Chlorhexidine in front of the mother or lack of communication between health care workers and women. A detailed validation analysis for Chlorhexidine application is published elsewhere [38].

We have considered “don’t know” replies for most yes/no survey questions as “no”, consistent with DHS reporting [40]. We found, however, for clinical interventions observed coverage was high among women who responded “don’t know”. While in our study, observed coverage of these clinical interventions was high among all newborns in these facilities, true coverage among women responding “don’t know” to these questions for home births or births in smaller facilities may not be as high. Survey-reported coverage of maternal and newborn care may have improved accuracy if “don’t know” responses are excluded from both numerators and denominators.

### Strengths and limitations

Strengths of this study include the large sample of more than 23,000 facility births (> 14,000 exit-surveys with women with vaginal births) across five high-burden facilities in three countries from sub-Saharan Africa and south Asia and direct observation by clinically trained researchers used as gold standard. Errors in data collection for observation were minimized by using a custom-built android application with time-stamping designed to reduce delay in recording events [52]. Data quality was promoted by refresher training and subsets of dual observation by supervisors for comparison [34]. While we did not base our assessment of validity on AUC cut-offs as our indicators were all binary (yes/no), we provide these calculations in Additional files 4 and 10.

This study's limitations included conducting the survey at the time of discharge from the hospital, in contrast to several years after birth as is often done in population-based surveys. As such, the recall bias will be minimized for our study, representing the best-case scenario and not the level of validity captured by population-based surveys. However, as surveys were conducted at the time of discharge, the busy clinical setting may have been distracting and women may have been in a hurry to return home, which may differ from the context of the population-based surveys occurring in a home setting. Some bias may have been introduced as > 5% of women were discharged before they were approached for interview. We also note that the results may not be representative of lower-level facilities since EN-BIRTH was conducted in five high-volume facilities. Additionally, observed coverage of care may have been higher due to the presence of the observer, further limiting generalisability and possibly altering women’s perception and recollection of care received [12]. In this paper we excluded the 6698 women who had caesarean sections. Since caesarean section affects both the practice of care and survey report, all our results for many of the 33 indicators would need to be split by caesarean section non-caesarean, adding even more complexity. These important analyses will be undertaken later.

The coverage of the indicators for treatment of presumed severe neonatal infection was reported from data extraction from individual case notes, as observation of admitted neonates for the whole hospital stay was not feasible. There is a possibility that a specific intervention was given but was not documented in the case notes. Despite having a large sample, there were still indicators with very high or low coverage that did not have enough observations in each column of the two-way table to report individual-level validity statistics. For those indicators, we did not report sensitivity, specificity, AUC and IF, and instead reported percent agreement [12]. The percentage agreement should be interpreted cautiously as there is the possibility of high percentage agreement for high sensitivity and low specificity of indicators that have high coverage. Additionally, high percentage agreement is also possible where an indicator has low sensitivity and high specificity with very low coverage.

Rates of caesarean sections are rising globally [53]. In our study, the caesarean section rate was 29% overall, and as high as 73% in one hospital, Azimpur BD. Women with vaginal births have different experiences from women undergoing caesareans and may experience more separation from their newborns. Caesarean birth negatively affected accuracy of survey-reported data [34–38]; thus this analysis has focused on vaginal birth. Further research of care and measurement among women with caesareans in this study is ongoing. Women with stillbirths were included in our survey, and coverage and measurement gaps for stillbirths are shown for specific indicators throughout this supplement series [35, 54]. The majority of women with stillbirths approached for survey consented to participate in and responded to questions on labour and birth [54], in line with other research involving women with stillbirths showing high survey completeness [55]. Women with stillbirths should be included in population-based surveys, particularly to inform action to end preventable stillbirths.

Further research is needed to understand if improving wording for some survey questions, particularly those related to clinical interventions or those with a timing component (i.e. early initiation of breastfeeding), may improve accuracy. Research on communication surrounding clinical interventions for newborn care, including small and sick newborns, is needed to understand factors contributing to accuracy of survey-reported coverage. More qualitative research regarding women’s understanding of and recall for questions related to timing, such as early breastfeeding and immediate drying, will allow us to improve question wording or indicator definitions. More process evaluation is required to better understand and improve aspects of surveys and survey burden.

## Conclusions

Population-based surveys remain an important source of generalisable maternal and newborn health information, especially where routine systems are not available. Among 33 indicators assessed, survey-reported birthweight measured and low birthweight classification performed well, however other clinical intervention questions and early initiation of breastfeeding performed poorly in survey-report. Further research is needed to see if differently phrased questions could lead to higher accuracy. While specific clinical interventions are not appropriate for surveys, contact indicators such as admission to a neonatal unit or a KMC ward may be a useful survey indicator option as a marker of care for small and sick newborns. Given that ~ 80% of births worldwide are now in facilities, investment in routine health management information systems could improve potential for tracking coverage of clinically focused maternal and newborn health interventions. Household surveys have numerous questions, and careful evidence-based measurement approaches should be applied to select and reject which indicators are best measured in surveys and/or routine systems based on impact and validity. Valid measurement is required to track scale-up of high-impact interventions and end preventable deaths of women and newborns.

## Supplementary Information

**Additional file 1.** Exit survey question wording of EN-BIRTH study compared to DHS/MICS question wording.

**Additional file 2.** List of indicators with denominator used.

**Additional file 3.** Definitions and formulas for validation metrics.

**Additional file 4.** Full validation analysis of the selected indicators in the EN-BIRTH study.

**Additional file 5.** STROBE checklist for cross-sectional studies.

**Additional file 6.** Characteristics of mother & baby- Labour & Delivery of EN-BIRTH study.

**Additional file 7.** Characteristics of mother & baby- Kangaroo mother care (KMC) of EN-BIRTH study.

**Additional file 8.** Characteristics of mother and baby- Neonatal Infection of EN-BIRTH study.

**Additional file 9.** Observer-assessed and survey-reported coverage by site.

**Additional file 10.** Full validation analysis of the selected indicators included in DHS/MICS (only yes vs no).

**Additional file 11.** Pooled analysis (random effects).

**Additional file 12.** Ethical approval of local institutional review boards for EN-BIRTH study.

## Data Availability

The datasets generated during and/or analysed during the current study are available on LSHTM Data Compass repository, https://datacompass.lshtm.ac.uk/955/.

## Abbreviations

AUC: Area under the receiver operating curve
BD: Bangladesh
PPV: Positive predictive value
NPV: Negative predictive value
IF: Inflation factor
CIFF: Children’s Investment Fund Foundation
DHS: The Demographic and Health Survey Program
ENAP: *Every Newborn* Action Plan now branded as *Every Newborn*
EN-BIRTH: *Every Newborn*-Birth Indicators Research Tracking in Hospitals study
icddr,b: International Centre for Diarrheal Disease Research, Bangladesh
IHI: Ifakara Health Institute, Tanzania
LMIC: Low-Middle Income Country
LSHTM: London School of Hygiene & Tropical Medicine
MUHAS: Muhimbili University of Health and Allied Sciences, Tanzania
MICS: Multiple Indicator Cluster Survey
NP: Nepal
TZ: Tanzania
BMV: Bag-mask-ventilation
UNICEF: United Nations Children's Fund
