# Supplementary material for: Survey of women’s report for 33 maternal and newborn indicators: EN-BIRTH multi-country validation study
Source: BMC Pregnancy Childbirth. 2021 Mar 26;21(Suppl 1):238. doi: 10.1186/s12884-020-03425-6 (PMC7995710; doi:10.1186/s12884-020-03425-6)
Supplement: Supplementary file 3 — Additional file 3. Definitions and formulas for validation metrics. [file 12884_2020_3425_MOESM3_ESM.pdf]

Every Newborn BIRTH multi-country validation study: informing measurement of coverage and quality of maternal and newborn care

Survey of women's report for 33 maternal & newborn indicators: EN-BIRTH multi-country validation study

Additional File 3: Definitions and formulas for validation metrics

Table 1. Table of definitions of validation metrics (Adapted from Day et al [1] and Munos et al [2])

TERM	DEFINITION	FORMULA*
**SENSITIVITY (SE)**	The proportion of individuals who truly received an intervention who were classified as having received the intervention by survey questions.	$\frac{a}{a+c}$ $\frac{TP}{TP+TN}$
**SPECIFICITY (SP)**	The proportion of individuals who truly did not receive an intervention who were classified as not having received the intervention by survey questions.	$\frac{d}{b+d}$ $\frac{TN}{FP+TN}$
**PERCENT AGREEMENT (ACCURACY)**	The proportion of individuals surveyed who were correctly classified as having received or not having received the intervention.	$\frac{a+d}{n}$ $\frac{TP+TN}{n}$
**VALIDITY RATIO**	The population-level validity of the measure using survey coverage and observed coverage.	$\frac{\text{Survey coverage}}{\text{Observed coverage}}$
**POSITIVE PREDICTIVE VALUE (PPV)**	The probability that an intervention reported as received has been observed as received	$\frac{\mathrm{a}}{\mathrm{a}+\mathrm{b}}$ $\frac{TP}{TP+FP}$
**NEGATIVE PREDICTIVE VALUE (NPV)**	The probability that an intervention not reported had been observed as not received	$\frac{d}{\mathrm{c}+\mathrm{d}}$ $\frac{TN}{FN+TN}$
**AREA UNDER THE RECEIVER OPERATING CHARACTERISTIC CURVE (AUC)**	The probability that a test will correctly classify a randomly selected set of one positive observation and one negative observation.	Calculated as the area under the curve of sensitivity plotted against (1 − specificity)
**INFLATION FACTOR (IF)**	The degree to which the indicator would be over or under-estimated by women's self-report using a ratio of the estimated survey-based coverage accounting for sensitivity and specificity to its true (observed) coverage.	$\frac{Observed\ coverage\ x\ (SE+SP-1)\ +\ (1-SP)}{Observed\ coverage}$

*Variables defined in Table 2

Table 2. Two-by-two validation table

		GOLD STANDARD		
		**Positive**	**Negative**	**Total**
**SURVEY MEASUREMENT**	**Positive**	a<br>True Positive (TP)	b<br>False Positive (FP)	a+b<br>TP+FP
	**Negative**	c<br>False Negative (FN)	d<br>True Negative (TN)	c+d<br>FN+TN
	**Total**	a+c<br>TP+TN	b+d<br>FP+FN	n

1. Day LT, Ruysen H, Gordeev VS, Gore-Langton GR, Boggs D, Cousens S, et al. “Every Newborn-BIRTH” protocol: observational study validating indicators for coverage and quality of maternal and newborn health care in Bangladesh, Nepal and Tanzania. J Glob Health. 2019;9:010902.

2. Munos MK, Blanc AK, Carter ED, Eisele TP, Gesuale S, Katz J, et al. Validation studies for population-based intervention coverage indicators: design, analysis, and interpretation. J Glob Health. 2018;8:020804.
